# Frameworks for Guiding the Selection of Digital Data Collection Tools Used in Clinical Trials: Protocol for a Systematic Review

**DOI:** 10.2196/78529

**Published:** 2026-01-22

**Authors:** Bavatharani Ananthesayanan, Kayode Philip Fadahunsi, Huanhuan Xiong, John O'Donoghue

**Affiliations:** 1 Department of Business Information Systems, University College Cork, College Road, Cork, T12K8AF, Ireland, 353 (021) 490 3000; 2 Department of Primary Care and Public Health, The School of Public Health, Imperial College London, London, England, United Kingdom; 3 Malawi eHealth Research Centre, University College Cork, Cork, Ireland; 4 School of Medicine, University College Dublin, Dublin, Ireland

**Keywords:** clinical trials, data collection tool, framework, digital health technology, health technology selection, digital data collection

## Abstract

**Background:**

Data collection is an essential aspect of clinical trials because it forms the basis of the scientific analysis that evaluates the performance and safety of interventions. With the wide variety of digital data collection tools available, decision-makers responsible for choosing the appropriate tools for clinical trials must exercise caution. There are numerous challenges that could impact data collection, and a careful selection of tools is necessary to ensure that they effectively support the trial. Therefore, an evidence-based framework is needed to support the selection of an appropriate digital data collection tool in clinical trials.

**Objective:**

This systematic review aims to develop an evidence-based framework for the selection of digital data collection tools for clinical trials.

**Methods:**

Bibliographic databases including IEEE Xplore, eAIS, PubMed, CINAHL, MEDLINE, Embase, ClinicalTrials.gov, Scopus, and Web of Science will be searched for published articles. Additionally, searches will be performed for publicly available gray literature from reputable institutions such as the United States Food and Drug Administration and World Health Organization. Studies should include a framework that is relevant to selecting digital data collection tools for clinical trials. Two reviewers will independently use Covidence to screen and review the articles to be included. Data related to the selection of digital data collection tools will be extracted. Thematic synthesis will be conducted to develop a new evidence-based framework to select digital data collection tools for clinical trials.

**Results:**

The review started in May 2025 and is expected to be completed in December 2025. The searches yielded 9151 studies, which were reduced to 4333 after the removal of duplicates using Covidence.

**Conclusions:**

There is a dearth of established frameworks to guide the selection of digital data collection tools for clinical trials. This review aims to develop an evidence-based framework to support technology decision-makers in identifying and selecting tools that are fit-for-purpose, ensuring they meet the specific needs of clinical research settings.

## Introduction

### Selection of Digital Data Collection Tools in Clinical Trials

There is a growing momentum toward digitizing human clinical trials as they become more complex [1,2] Nowadays, pharmaceutical and biotech companies turn to technological solutions to lower costs, streamline study management, keep study information current, and deliver new interventions [3,4]. Digital health technologies (DHTs) are used to accelerate trial startup and execution [5]. DHTs allow for the rapid collection of efficacy and safety data on interventions [6]. Data collection is a key component of clinical trials, as data form the basis of the scientific analysis that informs researchers regarding the performance of their interventions. Digital data collection tools are designed to provide a structured and consistent way of gathering information to reduce errors and bias, which can affect the validity and reliability of research findings [7]. When data collection tools are not properly selected in clinical trials, they can cause substantial disruptions to trial management and execution and hence delay the approval of the intervention by the regulatory bodies [8].

With the wide variety of digital data collection tools available, technology decision-makers responsible for selecting the appropriate tools for clinical trials must exercise caution. There are numerous challenges that may impact data collection, and careful selection is necessary to ensure the tools effectively support the trial [1]. The technology decision-makers need to consider the use of DHTs from a multistakeholder perspective to ensure the selected technology is fit-for-purpose. Clinical trials differ from other research settings due to their rigorous requirements for data quality, patient safety, and regulatory compliance. Credible patient data must be collected in accordance with the Good Clinical Practice (GCP) guidelines [9-11]. Additionally, clinical trials progress through distinct phases, each designed to address specific research questions, from early safety and dosing to large-scale efficacy and postmarketing surveillance [12]. Costs typically increase substantially in phase 2 and phase 3 trials due to the larger number of participants and the global scope of these studies, often reaching several million dollars [2]. These highlight the need for a dedicated framework to guide decision-makers in selecting the right technology to ensure both data integrity and patient safety are in full compliance with the GCP guidelines.

Although different frameworks have been developed for DHTs, none are specifically designed to support the selection of DHTs for data collection in clinical trials. For example, the American Psychiatric Association’s (APA) app hierarchical framework [13] has been adapted into a checklist to support the selection of DHTs for research purposes, as shown in Table 1 [8].

**Table 1.** Decision-making checklist for the selection of digital health technology based on the APA’s app hierarchical framework.

Domain	Items
Ethical principles	Informed consent process
Autonomy	How does the technology work? What personal data are produced? How is privacy managed? How is data stored and shared? Is bystander data captured?
Beneficence	Favorable risk/benefit analysis? Summary of known risks? Potential of unknown risks? Sound risk management plan? Rigorous study design?
Justice	Sample selection is appropriate?
Privacy and confidentiality	Are data encrypted? Who has data access? Is data security sound? Does HIPAA^a^ apply? What data are shared? Is there a privacy agreement?
Efficacy	Is there evidence that the product works? Has it been used with the study population?
Usability	Is the technology customizable, sustainable, and accessible? What is needed to use the technology?
Interoperability / data sharing	Is data available for postresearch access and sharing? Is there interoperability into electronic health record?

a HIPAA: Health Insurance Portability and Accountability Act.

However, the scope of the checklist is narrow as the APA’s hierarchical framework solely focuses on the evaluation of smartphone apps in clinical care [13] and does not address critical trial considerations, such as end point validity. Similarly, the NHS digital technology assessment criteria [10] focus on the use of DHTs in health and social care, with limited guidance on whether a DHT can reliably capture trial end points, align with clinical trial procedures and data collection plans, or maintain research-grade data integrity. In the same vein, the National Institute for Health and Care Excellence evidence standards framework delivers a high-level guidance on effectiveness and economic impact of DHTs according to risk [14], but it lacks concrete criteria for evaluating DHTs as instruments for research data collection. Nevertheless, these frameworks offer essential evaluation criteria and provide valuable insights into foundational elements that can inform the development of a trial-specific framework. Thus, there is a need to map the existing evaluation criteria and identify gaps related to trial-specific needs. This will inform the development of a robust, evidence-based framework to support technology decision-makers in making informed choices while upholding scientific rigor and regulatory compliance. Such a framework will ensure that the selected digital data collection tools are capable of accurately capturing trial end points, maintaining data integrity, and integrating seamlessly with study workflows.

### Objective

This systematic review aims to develop an evidence-based framework for the selection of digital data collection tools for clinical trials.

### Review Questions

This systematic review aims to address the following questions:

1. What frameworks are relevant to the selection of digital data collection tools in clinical trials?
2. What criteria in existing frameworks are relevant to selecting digital data collection tools in clinical trials?

## Methods

### Trial Registration

The systematic review will be presented in accordance with the Preferred Reporting Items for Systematic Reviews and Meta-Analyses for Protocol (PRISMA-P) checklist [15] and provided in Checklist 1. This protocol is registered with the PROSPERO database (CRD420250612895) [16]. Although this manuscript provides detailed information than the registry entry, there are no significant deviations.

### Ethical Considerations

Formal ethical approval is not required, as primary data will not be collected in this study. The findings from this systematic review will be published in a peer-reviewed journal and public gray literature.

### Eligibility Criteria

#### Inclusion Criteria

The inclusion criteria in this systematic review are as follows:

- Presence of a framework: in this systematic review, a framework is defined as a structured, evidence-informed set of principles, models, or guidelines that provides explicit guidance [17] for the evaluation and selection of DHT for data collection in clinical trials. A framework categorizes relevant characteristics and provides a structured way to evaluate them [7,18]. Ideally, these characteristics would be accompanied by a set of guiding questions specific to each criterion, as shown in Table 1, prompting technology decision-makers to consider critical factors in the selection process [18].
- Presence of criteria relevant to digital data collection tools
- Published studies and gray literature
- Studies written in the English language
- Availability of full-text articles

#### Exclusion Criteria

We have excluded publications based on the following criteria:

- Studies that do not have a framework for digital data collection tools
- Studies that are not available in full text
- Studies that are published in a non-English language

### Information Sources

The search will be conducted using the following bibliographic databases through the online library portal of University College Cork: IEEE Xplore, eAIS, PubMed, CINAHL, MEDLINE, Embase, ClinicalTrials.Gov, Scopus, and Web of Science. These databases were selected to provide a comprehensive coverage of digital and health care research information sources. Additionally, searches will be performed for publicly available gray literature from reputable institutions including the National Institute for Health and Care Excellence, Medicines & Healthcare products Regulatory Agency (MHRA), the Food and Drug Administration (FDA), the American Psychological Association, and the World Health Organization (WHO). Citation tracking of included studies using Google Scholar and a manual search of the references will also be conducted to identify other relevant studies. No date restrictions will be applied to ensure that no potentially relevant literature is inadvertently excluded. The search will include all qualitative, mixed methods, review, and consensus statement designs, including expert consensus papers and regulatory agency guidelines. This approach allows for a comprehensive synthesis of available evidence.

### Search Strategy

The search will be conducted using terms related to 3 key concepts: digital health, data collection, and framework. Terms related to each concept will be combined with “OR” and the search results for all the concepts will be combined using “AND” in the databases, as outlined in Textbox 1. Truncations and wildcards will be used to increase the sensitivity of the search. For institutional websites, searches will be conducted using the search terms such as “digital health framework,” “data collection framework,” “digital technology framework,” “digital health guideline,” “data collection guideline,” and “digital technology guideline.” Platform filters will be used to narrow the scope of the search. The full search strategy for all databases is presented in Multimedia Appendix 1.

Textbox 1. **Search strategy terms used for each concept.**

- System or technology terms**:** "eClinical," "e-clinical," "mHealth," "m-health," "eHealth," "e-health," "telehealth," "tele-health," "health information system*," "health information technolog*," "mobile health," "digital health," "digital technology*," "digital device*," "digital tool*," "digital system*"
- Data collection terms: "data acquisition," "data collection," "data capture," "data gathering," "information acquisition," "information collection," "information capture," "information gathering"
- Method or evaluation terms: "framework*," "checklist*," "guideline*," "model*," "criteria," "taxonomy"

### Data Management and Study Selection

Deduplication, selection of studies, and data extraction will be performed using Covidence [19]. The selection of studies will be performed in two stages: (1) screening of titles and abstracts and (2) full-text review. Two reviewers (BA and JO) will independently screen and review papers based on the eligibility criteria. Any disagreement during the study selection will be resolved by discussion between the two reviewers and by adjudication by a third reviewer (KPF). Where a framework or different versions of the same framework appear in different studies, all the studies will be included if they meet the eligibility criteria. The inclusion of multiple studies with the same framework will not affect the result, as thematic synthesis will address redundant and overlapping criteria. A PRISMA (Preferred Reporting Items for Systematic Reviews and Meta-Analyses) flow chart will be used to illustrate the study selection process.

### Data Extraction

Study details and information related to frameworks for selection of digital data collection tools will be extracted. The study details that will be extracted include authors, affiliation, country, year of publication, objective of the study, and study design. Information related to each framework to be extracted will include the following:

- How the framework was developed
- Criteria in the framework and their definition
- Type of digital data collection tools
- Stakeholders associated with the framework

Thus, the main outcomes from the review will include the following:

- Existing frameworks that are relevant to the selection of digital data collection tools for clinical trials
- The criteria that are used in the frameworks
- A new evidence-based framework, identifying criteria that are relevant to selecting digital data collection tools for clinical trials

### Quality Assessment

Given the qualitative nature of this systematic review, a formal risk of bias assessment will not be conducted. However, a quality assessment will be conducted using the Mixed Methods Appraisal Tool (MMAT) that allows the evaluation of different study designs [20]. The MMAT was selected for this review due to the anticipated methodological diversity of the included sources, such as qualitative studies, Delphi studies, and quantitative surveys on framework development. The MMAT provides a consistent approach for appraising qualitative, quantitative, and mixed methods designs, thereby ensuring systematic evaluation of all evidence.

### Data Synthesis

Thematic synthesis [21] will be used to create a new evidence-based framework for the selection of data collection tools for clinical trials. This process will be performed in three stages: (1) coding of the definitions of the criteria in existing frameworks; (2) development of descriptive themes; and (3) formation of analytical themes. The extracted data will be manually coded using the highlight function of Microsoft Word (Version 2511), as we envisaged that the volume of texts would be manageable. The purpose of this coding is to identify unique concepts within the definitions of the criteria from the selected frameworks. Codes will then be grouped on the basis of their similarities and differences, with a descriptive theme assigned to capture the meaning of each group of similar codes. Coding and generation of descriptive themes will be performed by 2 independent reviewers (BA and KPF), with disagreement resolved by mutual discussion or adjudication by a third reviewer (JO) when necessary to ensure reliable and rigorous synthesis. The analytical themes will be derived from the descriptive themes based on the interpretation of the reviewers. To minimize bias, each reviewer will first develop the analytical themes independently, after which all the reviewers will meet to finalize the analytical synthesis [18]. A new evidence-based framework will be developed based on the descriptive and analytical themes.

## Results

The review started in May 2025 and is expected to be completed in December 2025. The searches yielded 9151 studies, which were reduced to 4333 after the removal of duplicates using Covidence. Screening and full-text review of these studies will be performed to identify the final set that will be included for thematic synthesis.

## Discussion

### Anticipated Findings

The primary objective of this systematic review is to develop an evidence-based framework to guide the selection of digital data collection tools for clinical trials. The resulting framework is expected to highlight core considerations such as usability, interoperability, data quality, regulatory compliance, security, and adaptability to trial-specific needs. By synthesizing findings from a wide range of sources, the review aims to provide structured guidance for selecting tools that are fit-for-purpose and capable of meeting the unique requirements of clinical research settings. Ultimately, this framework will support more consistent, transparent, and evidence-informed decision-making in clinical trial data collection.

### Comparison to Prior Work

Existing frameworks have focused on specific technologies [8], health care delivery [10], or regulatory aspects of digital tools [14]. This systematic review will develop a comprehensive, structured framework specifically tailored for the selection of digital data collection tools in clinical trials. This framework will also provide actionable guidance that can be applied in a clinical trial setting.

### Strengths and Limitations

A strength of this review is its systematic and comprehensive search strategy across multiple bibliographic databases, combined with the inclusion of gray literature from reputable regulatory and health organizations. This approach increases the likelihood of capturing a broad range of perspectives relevant to tool selection. Another strength is the involvement of 2 independent reviewers in study selection and data synthesis to ensure a transparent and reproducible process.

However, restricting the review to only studies published in English may exclude relevant frameworks in other languages [22,23]. Translating non-English sources could alter the precise meaning of key definitions and concepts. Including only English language materials helps in ensuring that the original intent and nuance of terms are accurately captured. Furthermore, the reliance on published and publicly available literature may overlook valuable insights contained in private industry reports or unpublished evaluations [24].

### Implications for Policy, Practice, and Future Research

The anticipated framework has implications for the conduct of clinical trials. By offering structured guidance for tool selection, it may reduce inefficiencies, enhance data quality, and support regulatory compliance, ultimately improving the reliability and success of clinical research. Beyond its immediate utility, the framework could serve as a foundation for the development of best practice guidelines or policies to standardize the selection of digital data collection tools. Future research should focus on getting inputs from relevant stakeholders and validating the framework in clinical trial environments. Thus, an e-Delphi study is being planned with a group of stakeholders involved in clinical trials, including principal investigators, project leads, and technology vendors, to reach consensus on the relevance, completeness, and applicability of the framework criteria.

### Conclusions

This systematic review will develop an evidence-based framework to guide the selection of digital data collection tools. Thus, the framework will provide practical guidance to technology decision-makers to improve consistency, transparency, and effectiveness in the adoption of digital technologies in clinical trials, ultimately contributing to improved trial quality and efficiency.

## Supplementary material

10.2196/78529 Multimedia Appendix 1 Search strategy.

10.2196/78529 Checklist 1 PRISMA-P checklist.

## Abbreviations

DHT: digital health technology
FDA: Food and Drug Administration
GCP: Good Clinical Practice
MHRA: Medicines & Healthcare products Regulatory Agency
MMAT: Mixed Methods Appraisal Tool
PRISMA: Preferred Reporting Items for Systematic Reviews and Meta-Analyses
PRISMA-P: Preferred Reporting Items for Systematic Reviews and Meta-Analyses for Protocol
WHO: World Health Organization
