# Supplementary material for: Frameworks for Guiding the Selection of Digital Data Collection Tools Used in Clinical Trials: Protocol for a Systematic Review
Source: JMIR Res Protoc. 2026 Jan 22;15:e78529. doi: 10.2196/78529 (PMC12826635; doi:10.2196/78529)
Supplement: Multimedia Appendix 1 [file resprot-v15-e78529-s001.docx]

**Multimedia Appendix 1: Search Strategy**

Database: Web of Science

Search Data: 26May2025

| # | Search Query | Results |
| --- | --- | --- |
| 1 | (TI=(framework*)) OR AB=(framework*) | 2020928 |
| 2 | (TI=(checklist*)) OR AB=(checklist*) | 80595 |
| 3 | (TI=(guideline*)) OR AB=(guideline*) | 689507 |
| 4 | (TI=(model*)) OR AB=(model*) | 12189580 |
| 5 | (TI=(criteria)) OR AB=(criteria) | 1469199 |
| 6 | (TI=(taxonomy)) OR AB=(taxonomy) | 87217 |
| 7 | #1 OR #2 OR #3 OR #4 OR #5 OR #6 | 15124531 |
| 8 | (TI=("information gathering")) OR AB=("information gathering") | 4929 |
| 9 | (TI=("information capture")) OR AB=("information capture") | 452 |
| 10 | (TI=("information collection")) OR AB=("information collection") | 3994 |
| 11 | (TI=("information acquisition")) OR AB=("information acquisition") | 5413 |
| 12 | (TI=("data gathering")) OR AB=("data gathering") | 9079 |
| 13 | (TI=("data capture")) OR AB=("data capture") | 5848 |
| 14 | (TI=("data collection")) OR AB=("data collection") | 202154 |
| 15 | (TI=("data acquisition")) OR AB=("data acquisition") | 77657 |
| 16 | #8 OR #9 OR #10 OR #11 OR #12 OR #13 OR #14 OR #15 | 304244 |
| 17 | (TI=("digital system*")) OR AB=("digital system*") | 7948 |
| 18 | (TI=("digital tool*")) OR AB=("digital tool*") | 9419 |
| 19 | (TI=("digital device*")) OR AB=("digital device*") | 5086 |
| 20 | (TI=("digital technolog*")) OR AB=("digital technolog*") | 33909 |
| 21 | (TI=("digital health")) OR AB=("digital health") | 10178 |
| 22 | (TI=("mobile health")) OR AB=("mobile health") | 10005 |
| 23 | (TI=("health information technolog*")) OR AB=("health information technolog*") | 3439 |
| 24 | (TI=("health information system*")) OR AB=("health information system*") | 5754 |
| 25 | (TI=(tele-health)) OR AB=(tele-health) | 479 |
| 26 | (TI=(telehealth)) OR AB=(telehealth) | 16745 |
| 27 | (TI=(e-health)) OR AB=(e-health) | 7350 |
| 28 | (TI=(ehealth)) OR AB=(ehealth) | 6993 |
| 29 | (TI=(m-health)) OR AB=(m-health) | 1237 |
| 30 | (TI=(mhealth)) OR AB=(mhealth) | 8646 |
| 31 | ((TI=(e-clinical)) or AB=(e-clinical)) | 725 |
| 32 | ((TI=(eclinical)) or AB=(eclinical)) | 16 |
| 33 | #17 OR #18 OR #19 OR #20 OR #21 OR #22 OR #23 OR #24 OR #25 OR #26 OR #27 OR #28 OR #29 OR #30 OR #31 OR #32 | 115005 |
| 34 | #7 AND #16 AND #33 | 1630 |

Database: EBSCOhost CINAHL Plus with Full Text

Search Date: 26May2025

| **#** | **Query** | **Results** |
| --- | --- | --- |
| S34 | S31 AND S32 AND S33 | 332 |
| S33 | S25 OR S26 OR S27 OR S28 OR S29 OR S30 | 1,170,005 |
| S32 | S17 OR S18 OR S19 OR S20 OR S21 OR S22 OR S23 OR S24 | 53,051 |
| S31 | S1 OR S2 OR S3 OR S4 OR S5 OR S6 OR S7 OR S8 OR S9 OR S10 OR S11 OR S12 OR S13 OR S14 OR S15 OR S16 | 30,569 |
| S30 | XB taxonomy | 5,221 |
| S29 | XB criteria | 243,404 |
| S28 | XB model* | 704,804 |
| S27 | XB guideline* | 195,666 |
| S26 | XB checklist* | 30,592 |
| S25 | XB framework* | 124,674 |
| S24 | XB "information gathering" | 646 |
| S23 | XB "information capture" | 32 |
| S22 | XB "information collection" | 228 |
| S21 | XB "information acquisition" | 227 |
| S20 | XB "data gathering" | 1,164 |
| S19 | XB "data capture" | 1,192 |
| S18 | XB "data collection" | 48,460 |
| S17 | XB "data acquisition" | 1,526 |
| S16 | XB "digital system*" | 298 |
| S15 | XB "digital tool*" | 993 |
| S14 | XB "digital device*" | 470 |
| S13 | XB "digital technolog*" | 2,685 |
| S12 | XB "digital health" | 3,560 |
| S11 | XB "mobile health" | 3,138 |
| S10 | XB "health information technolog*" | 2,413 |
| S9 | XB "health information system*" | 2,283 |
| S8 | XB tele-health | 154 |
| S7 | XB telehealth | 9,587 |
| S6 | XB e-health | 1,881 |
| S5 | XB ehealth | 3,192 |
| S4 | XB m-health | 306 |
| S3 | XB mhealth | 3,009 |
| S2 | XB e-clinical | 44 |
| S1 | XB eclinical | 10 |

Database: EBSCOhost Medline

Search Date: 26May2025

| **#** | **Query** | **Results** |
| --- | --- | --- |
| S34 | S31 AND S32 AND S33 | 1,113 |
| S33 | S25 OR S26 OR S27 OR S28 OR S29 OR S30 | 5,903,686 |
| S32 | S17 OR S18 OR S19 OR S20 OR S21 OR S22 OR S23 OR S24 | 132,682 |
| S31 | S1 OR S2 OR S3 OR S4 OR S5 OR S6 OR S7 OR S8 OR S9 OR S10 OR S11 OR S12 OR S13 OR S14 OR S15 OR S16 | 61,340 |
| S30 | XB taxonomy | 31,224 |
| S29 | XB criteria | 907,993 |
| S28 | XB model* | 4,316,248 |
| S27 | XB guideline* | 572,432 |
| S26 | XB checklist* | 67,654 |
| S25 | XB framework* | 502,290 |
| S24 | XB "information gathering" | 1,484 |
| S23 | XB "information capture" | 113 |
| S22 | XB "information collection" | 826 |
| S21 | XB "information acquisition" | 964 |
| S20 | XB "data gathering" | 2,505 |
| S19 | XB "data capture" | 3,618 |
| S18 | XB "data collection" | 105,722 |
| S17 | XB "data acquisition" | 19,132 |
| S16 | XB "digital system*" | 1,296 |
| S15 | XB "digital tool*" | 2,937 |
| S14 | XB "digital device*" | 1,488 |
| S13 | XB "digital technolog*" | 7,256 |
| S12 | XB "digital health" | 9,186 |
| S11 | XB "mobile health" | 8,601 |
| S10 | XB "health information technolog*" | 3,820 |
| S9 | XB "health information system*" | 5,491 |
| S8 | XB tele-health | 240 |
| S7 | XB telehealth | 14,767 |
| S6 | XB e-health | 3,454 |
| S5 | XB ehealth | 5,275 |
| S4 | XB m-health | 718 |
| S3 | XB mhealth | 7,219 |
| S2 | XB e-clinical | 131 |
| S1 | XB eclinical | 15 |

Database: Embase

Search Date: 26May2025

| No. | Query | Results |
| --- | --- | --- |
| #34 | #31 AND #32 AND #33 | 1342 |
| #33 | #25 OR #26 OR #27 OR #28 OR #29 OR #30 | 7727442 |
| #32 | #17 OR #18 OR #19 OR #20 OR #21 OR #22 OR #23 OR #24 | 207917 |
| #31 | #1 OR #2 OR #3 OR #4 OR #5 OR #6 OR #7 OR #8 OR #9 OR #10 OR #11 OR #12 OR #13 OR #14 OR #15 OR #16 | 75450 |
| #30 | taxonomy:ab,ti | 31157 |
| #29 | criteria:ab,ti | 1396861 |
| #28 | model*:ab,ti | 5442846 |
| #27 | guideline*:ab,ti | 875686 |
| #26 | checklist*:ab,ti | 90617 |
| #25 | framework*:ab,ti | 544399 |
| #24 | 'information gathering':ab,ti | 1980 |
| #23 | 'information capture':ab,ti | 145 |
| #22 | 'information collection':ab,ti | 1079 |
| #21 | 'information acquisition':ab,ti | 994 |
| #20 | 'data capture':ab,ti | 6870 |
| #19 | 'data capture':ab,ti | 6870 |
| #18 | 'data collection':ab,ti | 175725 |
| #17 | 'data acquisition':ab,ti | 23502 |
| #16 | 'digital system*':ab,ti | 1654 |
| #15 | 'digital tool*':ab,ti | 3557 |
| #14 | 'digital device*':ab,ti | 1841 |
| #13 | 'digital technolog*':ab,ti | 7805 |
| #12 | 'digital health':ab,ti | 9906 |
| #11 | 'mobile health':ab,ti | 9148 |
| #10 | 'health information technolog*':ab,ti | 4135 |
| #9 | 'health information system*':ab,ti | 6973 |
| #8 | 'tele health':ab,ti | 635 |
| #7 | telehealth:ab,ti | 20279 |
| #6 | 'e health':ab,ti | 5402 |
| #5 | ehealth:ab,ti | 10224 |
| #4 | 'm health':ab,ti | 1045 |
| #3 | mhealth:ab,ti | 8562 |
| #2 | 'e clinical':ab,ti | 1173 |
| #1 | eclinical:ab,ti | 89 |

Database: PubMed

Search Date: 26May2025

| Search number | Search Details | Results |
| --- | --- | --- |
| 34 | ("eclinical"[Title/Abstract] OR "e-clinical"[Title/Abstract] OR "mhealth"[Title/Abstract] OR "m-health"[Title/Abstract] OR "ehealth"[Title/Abstract] OR "e-health"[Title/Abstract] OR "telehealth"[Title/Abstract] OR "tele-health"[Title/Abstract] OR "health information system*"[Title/Abstract] OR "health information technolog*"[Title/Abstract] OR "mobile health"[Title/Abstract] OR "digital health"[Title/Abstract] OR "digital technolog*"[Title/Abstract] OR "digital device*"[Title/Abstract] OR "digital tool*"[Title/Abstract] OR "digital system*"[Title/Abstract]) AND ("data acquisition"[Title/Abstract] OR "data collection"[Title/Abstract] OR "data capture"[Title/Abstract] OR "data gathering"[Title/Abstract] OR "information acquisition"[Title/Abstract] OR "information collection"[Title/Abstract] OR "information capture"[Title/Abstract] OR "information gathering"[Title/Abstract]) AND ("framework*"[Title/Abstract] OR "checklist*"[Title/Abstract] OR "guideline*"[Title/Abstract] OR "model*"[Title/Abstract] OR "criteria"[Title/Abstract] OR "taxonomy"[Title/Abstract]) | 1,481 |
| 33 | "framework*"[Title/Abstract] OR "checklist*"[Title/Abstract] OR "guideline*"[Title/Abstract] OR "model*"[Title/Abstract] OR "criteria"[Title/Abstract] OR "taxonomy"[Title/Abstract] | 5,920,453 |
| 32 | "data acquisition"[Title/Abstract] OR "data collection"[Title/Abstract] OR "data capture"[Title/Abstract] OR "data gathering"[Title/Abstract] OR "information acquisition"[Title/Abstract] OR "information collection"[Title/Abstract] OR "information capture"[Title/Abstract] OR "information gathering"[Title/Abstract] | 150,062 |
| 31 | "eclinical"[Title/Abstract] OR "e-clinical"[Title/Abstract] OR "mhealth"[Title/Abstract] OR "m-health"[Title/Abstract] OR "ehealth"[Title/Abstract] OR "e-health"[Title/Abstract] OR "telehealth"[Title/Abstract] OR "tele-health"[Title/Abstract] OR "health information system*"[Title/Abstract] OR "health information technolog*"[Title/Abstract] OR "mobile health"[Title/Abstract] OR "digital health"[Title/Abstract] OR "digital technolog*"[Title/Abstract] OR "digital device*"[Title/Abstract] OR "digital tool*"[Title/Abstract] OR "digital system*"[Title/Abstract] | 77,693 |
| 30 | "taxonomy"[Title/Abstract] | 44,995 |
| 29 | "criteria"[Title/Abstract] | 843,164 |
| 28 | "model*"[Title/Abstract] | 4,367,311 |
| 27 | "guideline*"[Title/Abstract] | 566,353 |
| 26 | "checklist*"[Title/Abstract] | 68,980 |
| 25 | "framework*"[Title/Abstract] | 506,859 |
| 24 | "information gathering"[Title/Abstract] | 1,530 |
| 23 | "information capture"[Title/Abstract] | 113 |
| 22 | "information collection"[Title/Abstract] | 821 |
| 21 | "information acquisition"[Title/Abstract] | 973 |
| 20 | "data gathering"[Title/Abstract] | 2,521 |
| 19 | "data capture"[Title/Abstract] | 3,674 |
| 18 | "data collection"[Title/Abstract] | 123,143 |
| 17 | "data acquisition"[Title/Abstract] | 19,063 |
| 16 | "digital system*"[Title/Abstract] | 1,305 |
| 15 | "digital tool*"[Title/Abstract] | 3,087 |
| 14 | "digital device*"[Title/Abstract] | 1,551 |
| 13 | "digital technolog*"[Title/Abstract] | 8,108 |
| 12 | "digital health"[Title/Abstract] | 13,783 |
| 11 | "mobile health"[Title/Abstract] | 11,536 |
| 10 | "health information technolog*"[Title/Abstract] | 4,682 |
| 9 | "health information system*"[Title/Abstract] | 6,052 |
| 8 | "tele-health"[Title/Abstract] | 366 |
| 7 | "telehealth"[Title/Abstract] | 18,942 |
| 6 | "e-health"[Title/Abstract] | 5,320 |
| 5 | "ehealth"[Title/Abstract] | 12,075 |
| 4 | "m-health"[Title/Abstract] | 1,107 |
| 3 | "mhealth"[Title/Abstract] | 12,751 |
| 2 | "e-clinical"[Title/Abstract] | 698 |
| 1 | "eclinical"[Title/Abstract] | 26 |

Database: Scopus

Search Date: 26May2025

| 34 | ( ( ( TITLE ( eclinical ) OR ABS ( eclinical ) ) ) OR ( ( TITLE ( e-clinical ) OR ABS ( e-clinical ) ) ) OR ( ( TITLE ( mhealth ) OR ABS ( mhealth ) ) ) OR ( ( TITLE ( m-health ) OR ABS ( m-health ) ) ) OR ( ( TITLE ( ehealth ) OR ABS ( ehealth ) ) ) OR ( ( TITLE ( e-health ) OR ABS ( e-health ) ) ) OR ( ( TITLE ( telehealth ) OR ABS ( telehealth ) ) ) OR ( ( TITLE ( tele-health ) OR ABS ( tele-health ) ) ) OR ( ( TITLE ( "health information system*" ) OR ABS ( "health information system*" ) ) ) OR ( ( TITLE ( "health information technolog*" ) OR ABS ( "health information technolog*" ) ) ) OR ( ( TITLE ( "mobile health" ) OR ABS ( "mobile health" ) ) ) OR ( ( TITLE ( "digital health" ) OR ABS ( "digital health" ) ) ) OR ( ( TITLE ( "digital technolog*" ) OR ABS ( "digital technolog*" ) ) ) OR ( ( TITLE ( "digital device*" ) OR ABS ( "digital device*" ) ) ) OR ( ( TITLE ( "digital tool*" ) OR ABS ( "digital tool*" ) ) ) OR ( ( TITLE ( "digital system*" ) OR ABS ( "digital system*" ) ) ) ) AND ( ( ( TITLE ( "data acquisition" ) OR ABS ( "data acquisition" ) ) ) OR ( ( TITLE ( "data collection" ) OR ABS ( "data collection" ) ) ) OR ( ( TITLE ( "data capture" ) OR ABS ( "data capture" ) ) ) OR ( ( TITLE ( "data gathering" ) OR ABS ( "data gathering" ) ) ) OR ( ( TITLE ( "information acquisition" ) OR ABS ( "information acquisition" ) ) ) OR ( ( TITLE ( "information collection" ) OR ABS ( "information collection" ) ) ) OR ( ( TITLE ( "information capture" ) OR ABS ( "information capture" ) ) ) OR ( ( TITLE ( "information gathering" ) OR ABS ( "information gathering" ) ) ) ) AND ( ( ( TITLE ( framework* ) OR ABS ( framework* ) ) ) OR ( ( TITLE ( checklist* ) OR ABS ( checklist* ) ) ) OR ( ( TITLE ( guideline* ) OR ABS ( guideline* ) ) ) OR ( ( TITLE ( model* ) OR ABS ( model* ) ) ) OR ( ( TITLE ( taxonomy ) OR ABS ( taxonomy ) ) ) OR ( ( TITLE ( criteria ) OR ABS ( criteria ) ) ) ) | 2,327 |
| --- | --- | --- |
| 33 | ( ( TITLE ( framework* ) OR ABS ( framework* ) ) ) OR ( ( TITLE ( checklist* ) OR ABS ( checklist* ) ) ) OR ( ( TITLE ( guideline* ) OR ABS ( guideline* ) ) ) OR ( ( TITLE ( model* ) OR ABS ( model* ) ) ) OR ( ( TITLE ( taxonomy ) OR ABS ( taxonomy ) ) ) OR ( ( TITLE ( criteria ) OR ABS ( criteria ) ) ) | 20,173,821 |
| 32 | ( ( TITLE ( "data acquisition" ) OR ABS ( "data acquisition" ) ) ) OR ( ( TITLE ( "data collection" ) OR ABS ( "data collection" ) ) ) OR ( ( TITLE ( "data capture" ) OR ABS ( "data capture" ) ) ) OR ( ( TITLE ( "data gathering" ) OR ABS ( "data gathering" ) ) ) OR ( ( TITLE ( "information acquisition" ) OR ABS ( "information acquisition" ) ) ) OR ( ( TITLE ( "information collection" ) OR ABS ( "information collection" ) ) ) OR ( ( TITLE ( "information capture" ) OR ABS ( "information capture" ) ) ) OR ( ( TITLE ( "information gathering" ) OR ABS ( "information gathering" ) ) ) | 478,033 |
| 31 | ( ( TITLE ( eclinical ) OR ABS ( eclinical ) ) ) OR ( ( TITLE ( e-clinical ) OR ABS ( e-clinical ) ) ) OR ( ( TITLE ( mhealth ) OR ABS ( mhealth ) ) ) OR ( ( TITLE ( m-health ) OR ABS ( m-health ) ) ) OR ( ( TITLE ( ehealth ) OR ABS ( ehealth ) ) ) OR ( ( TITLE ( e-health ) OR ABS ( e-health ) ) ) OR ( ( TITLE ( telehealth ) OR ABS ( telehealth ) ) ) OR ( ( TITLE ( tele-health ) OR ABS ( tele-health ) ) ) OR ( ( TITLE ( "health information system*" ) OR ABS ( "health information system*" ) ) ) OR ( ( TITLE ( "health information technolog*" ) OR ABS ( "health information technolog*" ) ) ) OR ( ( TITLE ( "mobile health" ) OR ABS ( "mobile health" ) ) ) OR ( ( TITLE ( "digital health" ) OR ABS ( "digital health" ) ) ) OR ( ( TITLE ( "digital technolog*" ) OR ABS ( "digital technolog*" ) ) ) OR ( ( TITLE ( "digital device*" ) OR ABS ( "digital device*" ) ) ) OR ( ( TITLE ( "digital tool*" ) OR ABS ( "digital tool*" ) ) ) OR ( ( TITLE ( "digital system*" ) OR ABS ( "digital system*" ) ) ) | 170,014 |
| 30 | ( TITLE ( criteria ) OR ABS ( criteria ) ) | 2,054,469 |
| 29 | ( TITLE ( taxonomy ) OR ABS ( taxonomy ) ) | 112,888 |
| 28 | ( TITLE ( model* ) OR ABS ( model* ) ) | 16,075,401 |
| 27 | ( TITLE ( guideline* ) OR ABS ( guideline* ) ) | 951,160 |
| 26 | ( TITLE ( checklist* ) OR ABS ( checklist* ) ) | 103,091 |
| 25 | ( TITLE ( framework* ) OR ABS ( framework* ) ) | 2,796,664 |
| 24 | ( TITLE ( "information gathering" ) OR ABS ( "information gathering" ) ) | 7,982 |
| 23 | ( TITLE ( "information capture" ) OR ABS ( "information capture" ) ) | 786 |
| 22 | ( TITLE ( "information collection" ) OR ABS ( "information collection" ) ) | 7,547 |
| 21 | ( TITLE ( "information acquisition" ) OR ABS ( "information acquisition" ) ) | 8,283 |
| 20 | ( TITLE ( "data gathering" ) OR ABS ( "data gathering" ) ) | 16,749 |
| 19 | ( TITLE ( "data capture" ) OR ABS ( "data capture" ) ) | 8,947 |
| 18 | ( TITLE ( "data collection" ) OR ABS ( "data collection" ) ) | 308,434 |
| 17 | ( TITLE ( "data acquisition" ) OR ABS ( "data acquisition" ) ) | 128,473 |
| 16 | ( TITLE ( "digital system*" ) OR ABS ( "digital system*" ) ) | 15,447 |
| 15 | ( TITLE ( "digital tool*" ) OR ABS ( "digital tool*" ) ) | 14,925 |
| 14 | ( TITLE ( "digital device*" ) OR ABS ( "digital device*" ) ) | 8,095 |
| 13 | ( TITLE ( "digital technolog*" ) OR ABS ( "digital technolog*" ) ) | 60,019 |
| 12 | ( TITLE ( "digital health" ) OR ABS ( "digital health" ) ) | 12,408 |
| 11 | ( TITLE ( "mobile health" ) OR ABS ( "mobile health" ) ) | 12,317 |
| 10 | ( TITLE ( "health information technolog*" ) OR ABS ( "health information technolog*" ) ) | 5,057 |
| 9 | ( TITLE ( "health information system*" ) OR ABS ( "health information system*" ) ) | 7,926 |
| 8 | ( TITLE ( tele-health ) OR ABS ( tele-health ) ) | 568 |
| 7 | ( TITLE ( telehealth ) OR ABS ( telehealth ) ) | 18,062 |
| 6 | ( TITLE ( e-health ) OR ABS ( e-health ) ) | 11,253 |
| 5 | ( TITLE ( ehealth ) OR ABS ( ehealth ) ) | 8,916 |
| 4 | ( TITLE ( m-health ) OR ABS ( m-health ) ) | 1,752 |
| 3 | ( TITLE ( mhealth ) OR ABS ( mhealth ) ) | 10,158 |
| 2 | ( TITLE ( e-clinical ) OR ABS ( e-clinical ) ) | 969 |
| 1 | ( TITLE ( eclinical ) OR ABS ( eclinical ) ) | 30 |

Database: ClinicalTrial.Gov

Search Date: 26May2025

| No | Queries | Results |
| --- | --- | --- |
| 4 | (eclinical OR e-clincal OR mhealth OR m-health OR ehealth OR e-health OR telehealth OR tele-health OR "health information system*" OR "health information technolog*" OR "mobile health" OR "digital health" OR "digital technolog*" OR "digital device*" OR "digital tool*" OR "digtial system*") AND ("data acquisition" OR "data collection" OR "data capture" OR "data gathering" OR "information acquisition" OR "information collection" OR "information capture" OR "information gathering") AND (framework* OR checklist* OR guideline* OR model* OR criteria OR taxonomy) | 704 |
| 3 | framework* OR checklist* OR guideline* OR model* OR criteria OR taxonomy | 128,264 |
| 2 | ("data acquisition" OR "data collection" OR "data capture" OR "data gathering" OR "information acquisition" OR "information collection" OR "information capture" OR "information gathering") | 8,843 |
| 1 | (eclinical OR e-clincal OR mhealth OR m-health OR ehealth OR e-health OR telehealth OR tele-health OR "health information system*" OR "health information technolog*" OR "mobile health" OR "digital health" OR "digital technolog*" OR "digital device*" OR "digital tool*" OR "digtial system*") | 41,957 |

Database: eAIS

Search Date: 26May2025

| No. | Search Query | Results |
| --- | --- | --- |
| 2 | abstract:( eclinical OR e-clinical OR mhealth OR m-health OR ehealth OR e-health OR telehealth OR tele-health OR "health information system*" OR "health information technolog*" OR "mobile health" OR "digital health" OR "digital technolog*" OR "digital device*" OR "digital tool*" OR "digital system*" ) AND abstract:( "data acquisition" OR "data collection" OR "data capture" OR "data gathering" OR "information acquisition" OR "information collection" OR "information capture" OR "information gathering" ) AND abstract:( framework* OR checklist* OR guideline* OR model* OR criteria OR taxonomy ) | 39 |
| 1 | title:( eclinical OR e-clinical OR mhealth OR m-health OR ehealth OR e-health OR telehealth OR tele-health OR "health information system*" OR "health information technolog*" OR "mobile health" OR "digital health" OR "digital technolog*" OR "digital device*" OR "digital tool*" OR "digital system*" ) AND title:( "data acquisition" OR "data collection" OR "data capture" OR "data gathering" OR "information acquisition" OR "information collection" OR "information capture" OR "information gathering" ) AND title:( framework* OR checklist* OR guideline* OR model* OR criteria OR taxonomy ) | 0 |

Database: IEEE

Search Date: 26May2025

| No. | Search Query | Results |
| --- | --- | --- |
| 4 | ("Document Title":eclinical OR "Document Title":e-clinical) AND ("Document Title":"data acquisition" OR "Document Title":"data collection" OR "Document Title":"data capture" OR "Document Title":"data gathering" OR "Document Title":"information acquisition" OR "Document Title":"information collection" OR "Document Title":"information capture" OR "Document Title":"information gathering") AND ("Document Title":framework* OR "Document Title":checklist* OR "Document Title":guideline* OR "Document Title":model* OR "Document Title":criteria OR "Document Title":taxonomy) | 0 |
| 3 | ("Abstract":eclinical OR "Abstract":e-clinical) AND ("Abstract":"data acquisition" OR "Abstract":"data collection" OR "Abstract":"data capture" OR "Abstract":"data gathering" OR "Abstract":"information acquisition" OR "Abstract":"information collection" OR "Abstract":"information capture" OR "Abstract":"information gathering") AND ("Abstract":framework* OR "Abstract":checklist* OR "Abstract":guideline* OR "Abstract":model* OR "Abstract":criteria OR "Abstract":taxonomy) | 0 |
| 2 | ("Document Title":mhealth OR "Document Title":m-health OR "Document Title":ehealth OR "Document Title":e-health OR "Document Title":telehealth OR "Document Title":tele-health OR "Document Title":"health information system*" OR "Document Title":"health information technolog*" OR "Document Title":"mobile health" OR "Document Title":"digital health" OR "Document Title":"digital technolog*" OR "Document Title":"digital device*" OR "Document Title":"digital tool*" OR "Document Title":"digital system*") AND ("Document Title":"data acquisition" OR "Document Title":"data collection" OR "Document Title":"data capture" OR "Document Title":"data gathering" OR "Document Title":"information acquisition" OR "Document Title":"information collection" OR "Document Title":"information capture" OR "Document Title":"information gathering") AND ("Document Title":framework* OR "Document Title":checklist* OR "Document Title":guideline* OR "Document Title":model* OR "Document Title":criteria OR "Document Title":taxonomy) | 1 |
| 1 | ("Abstract":mhealth OR "Abstract":m-health OR "Abstract":ehealth OR "Abstract":e-health OR "Abstract":telehealth OR "Abstract":tele-health OR "Abstract":"health information system*" OR "Abstract":"health information technolog*" OR "Abstract":"mobile health" OR "Abstract":"digital health" OR "Abstract":"digital technolog*" OR "Abstract":"digital device*" OR "Abstract":"digital tool*" OR "Abstract":"digital system*") AND ("Abstract":"data acquisition" OR "Abstract":"data collection" OR "Abstract":"data capture" OR "Abstract":"data gathering" OR "Abstract":"information acquisition" OR "Abstract":"information collection" OR "Abstract":"information capture" OR "Abstract":"information gathering") AND ("Abstract":framework* OR "Abstract":checklist* OR "Abstract":guideline* OR "Abstract":model* OR "Abstract":criteria OR "Abstract":taxonomy) | 170 |
